# Comparing survival with vitamin K antagonists, low-molecular-weight heparin, and direct oral anticoagulants in patients with cancer—a systematic review and meta-analysis

**DOI:** 10.1016/j.rpth.2025.103268

**Published:** 2025-11-19

**Authors:** Vasiliki Xirou, Anika Patel, Maria Fernanda Albuja Altamirano, Rishabh Singh, Jason Gusdorf, Kevin Barnum, Megan McNichol, Justine Ryu, Jeffrey I. Zwicker, Thita Chiasakul, Rushad Patell

**Affiliations:** 1 Department of Medicine, Mount Auburn Hospital, Harvard Medical School, Cambridge, Massachusetts, USA; 2 Creighton University School of Medicine, Omaha, Nebraska, USA; 3 Department of Medicine, Lincoln Hospital, New York, New York, USA; 4 Department of Medicine, University of Illinois College of Medicine, Peoria, Illinois, USA; 5 Department of Medicine, Beth Israel Deaconess Medical Center, Harvard Medical School, Boston, Massachusetts, USA; 6 Division of Hematology and Hematologic Malignancies, Department of Medicine, Beth Israel Deaconess Medical Center, Harvard Medical School, Boston, Massachusetts, USA; 7 Department of Information Technology/Division of Knowledge Services, Beth Israel Lahey Health, Cambridge, Massachusetts, USA; 8 Department of Medicine, Section of Hematology, Yale School of Medicine, New Haven, Connecticut, USA; 9 Hematology Service, Department of Medicine, Memorial Sloan Kettering Cancer Center, New York, New York, USA; 10 Weill Cornell Medical College, New York, New York, USA; 11 Center of Excellence in Translational Hematology, Division of Hematology, Department of Medicine, Faculty of Medicine, Chulalongkorn University and King Chulalongkorn Memorial Hospital, Thai Red Cross Society, Bangkok, Thailand

**Keywords:** anticoagulants, apixaban, edoxaban, hemorrhage, heparin, low-molecular-weight, mortality, rivaroxaban, thrombosis, venous thrombosis, warfarin

## Abstract

**Background:**

Venous thromboembolism (VTE) is a frequent complication in malignancy. Low-molecular-weight heparins and direct oral anticoagulants have replaced vitamin K antagonists (VKAs) as the standard of care for cancer-associated VTE. Nonetheless, clinical trials have not established a survival benefit of these agents compared with VKA.

**Objectives:**

We conducted a systematic review and meta-analysis to compare survival in cancer patients receiving VKA vs other anticoagulants.

**Methods:**

We searched Embase, Web of Science, PubMed, ClinicalTrials.gov, and Cochrane from inception until April 10, 2025, focusing on the use of VKA and non-VKA in cancer patients. Primary outcome was mortality and secondary outcomes included thromboembolism and bleeding.

**Results:**

Of 11,198 studies screened, 14 studies (70,025 patients) were included. VKA were associated with lower mortality than non-VKA in observational studies (odds ratio [OR], 0.84; 95% CI, 0.78-0.91; *I*^2^ = 81%; *n* = 6 studies) but not in randomized controlled trials (OR, 0.99; 95% CI, 0.86-1.13; *I*^2^ = 0%; *n* = 8 studies). In subgroup analysis, follow-up period of >6 months (OR, 0.85; 95% CI, 0.79-0.92; *I*^2^ = 75%), solid malignancies (OR, 0.81; 95% CI, 0.75-0.88; *I*^2^ = 78%), and indication of VTE only (OR, 0.89; 95% CI, 0.83-0.96; *I*^2^ = 42%) demonstrated improved survival with VKA.

**Conclusion:**

The use of VKA was associated with lower mortality than non-VKA anticoagulation in patients with cancer in observational studies but not in randomized trials. The analysis was limited by high heterogeneity, which must be considered when interpreting results.

## Essentials

• Venous thromboembolism is a frequent complication of malignancy, and although low-molecular-weight heparin and direct oral anticoagulants have replaced vitamin K antagonists as standard therapy, their effect on overall survival remains uncertain.
• We conducted a systematic review and random-effects meta-analysis comparing vitamin K antagonists with low-molecular-weight heparin and direct oral anticoagulants in patients with cancer, with all-cause mortality as the primary outcome.
• Vitamin K antagonist use was associated with lower mortality in observational studies but not in randomized controlled trials, with substantial statistical heterogeneity across studies.
• The survival signal was most evident with longer follow-up, in solid tumors, and when anticoagulation was indicated for venous thromboembolism alone; however, heterogeneity limits causal inference and bleeding risk was similar across treatments.

## Graphical abstract

**Figure:**
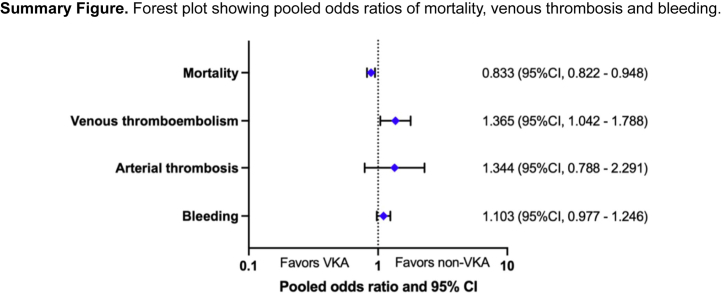

## 1 Introduction

Patients with cancer face up to a 7-fold increased risk of venous thromboembolism (VTE) compared with the general population [1]. Thromboembolic events contribute not only to increased morbidity and mortality but also to higher health care costs [2,3]. Since their approval for clinical use in the United States in 1954, warfarin and other vitamin K antagonists (VKAs) remained the standard anticoagulant therapy for nearly half a century. With the introduction of low-molecular-weight heparins (LMWHs) into clinical practice, subsequent studies demonstrated their superiority over VKAs in reducing VTE incidence, with a lower risk of bleeding complications [4,5]. In the years that followed, clinical trials comparing LMWH with direct oral anticoagulants (DOACs) demonstrated that DOACs were noninferior in both efficacy and safety [[6], [7], [8], [9]]. Today, DOACs, alongside LMWH, are the standard of care anticoagulant in most recent clinical guidelines [10,11]. In this evolving treatment landscape, VKAs are now considered an alternative option, reserved for cases where LMWH or DOACs are deemed unsuitable.

Although the benefits of LMWH and DOACs in preventing recurrent VTE and minimizing bleeding complications are well established, anticoagulation decisions in patients with cancer require consideration of multiple additional factors. These include patient preference, quality of life, potential interactions with anticancer therapies, treatment adherence, and cost [[12], [13], [14]]. However, unarguably overall survival (OS) remains the most meaningful measure of clinical benefit.

Antineoplastic and antimetastatic effects have been linked to the use of VKAs since the 1970s [15,16]. Despite an expanding body of evidence since, from basic science, clinical trials, and epidemiological studies, whether warfarin confers clinically meaningful antineoplastic benefits independent of its anticoagulant properties remains unclear. We conducted a systematic review and metanalyzes of current literature to assess the impact of VKA compared with alternate anticoagulants on mortality in patients with cancer.

## 2 Methods

### 2.1 Data sources and search strategy

A systematic literature search was performed using the following databases: Embase, Cochrane Library, PubMed/MEDLINE, Web of Science, and ClinicalTrials.gov from inception to April 10, 2025 (Supplementary Table 1). The study protocol was registered on the International Prospective Register of Systematic Reviews (PROSPERO, CRD42023488569). The Preferred Reporting Items for Systematic Reviews and Meta-analyses (PRISMA) guidelines were followed. Depending on the databases being used, appropriate subject headings and keywords were added as needed. Only English-language studies were included. The knowledge specialist (M.M.) used the PubMed ReMiner tool to extract keywords and index terms to develop the initial search strategy and translated the search strategy using the Polyglot Search Accelerator.

### 2.2 Study selection

Randomized controlled trials (RCTs), retrospective or prospective observational studies, and case series involving at least 10 patients were included in this metanalysis. We focused on adult patients with solid or hematologic malignancies who received treatment with VKA, LMWH, or DOACs for the indication of atrial fibrillation (AF) or arterial/venous thrombosis.

Studies were excluded if they lacked a treatment or comparison group involving VKAs or did not report mortality outcomes. Editorials, commentaries, case reports, and case series with fewer than 10 patients were also excluded. To be included, studies were required to report at least mortality separately for each anticoagulant treatment group.

Five reviewers (V.X., A.P., M.F.A.A., R.S., and J.G.) independently screened titles and abstracts through the Covidence online platform. Discrepancies were resolved by consultation with a third reviewer. Following the initial screening, full-text articles were independently assessed for eligibility by 2 reviewers (V.X., and A.P.).

### 2.3 Data abstraction

Five reviewers independently and systematically extracted data on study characteristics and outcomes of interest in duplicate. The primary outcome of this review was all-cause mortality. Secondary outcomes included VTE, defined as a composite of pulmonary embolism, deep vein thrombosis, and thrombosis in other sites (such as splanchnic veins and cerebral venous sinuses); arterial thrombosis; and bleeding. Of the studies that included bleeding as an outcome, most defined episodes as either clinically relevant nonmajor bleeding or major bleeding as described by previous publications [17,18]. Some studies also reported minor bleeding episodes defined as those not filling criteria for major bleeding.

### 2.4 Quality and risk of bias assessment

Risk of bias was assessed using the Cochrane Collaboration’s Risk of Bias tool version 2 for RCTs [19], and an adapted version of the Methodological Index for Non–Randomized Studies (MINORS) criteria [20] for observational comparative studies. Publication bias was assessed using visual inspection of funnel plots for asymmetry and quantitatively via Egger regression test for mortality outcome. A *P* value of <.10 for Egger test was considered indicative of small-study effects suggestive of potential publication bias.

### 2.5 Data synthesis and analysis

Data analysis was performed using Comprehensive Meta-analysis (version 3.0). Random-effects meta-analysis was conducted using the DerSimonian and Laird method to account for between-study heterogeneity. We calculated standardized event rates (incidence rates per patient-month [PM] of follow-up) to address potential bias from variation in follow-up durations across studies and treatment arms. For that reason, this analysis was limited to studies that reported either the mean or median follow-up duration. It is important to note that individual-level follow-up durations were not available; therefore, this approach provides an approximate estimate, which has been previously applied in the literature [[21], [22], [23]]. For each study, total PMs of follow-up were calculated by multiplying the reported mean or median follow-up time by the number of patients in each treatment group. Pooled outcome rates (calculated per 100 PMs) and corresponding 95% CIs were then estimated. These pooled event rates were analyzed for each anticoagulant when data on specific numbers of patients treated with VKA, LMWH, or DOAC were available.

A random-effects model was used to pool the odds ratios (OR) of these standardized event rates across studies, allowing for a within-study comparison that accounts for heterogeneity in patient populations, follow-up duration, and study-level weighting. The ORs were calculated for VKA vs other anticoagulants (LMWH or DOACs), VKA vs LMWH, and VKA vs DOACs. Heterogeneity was assessed using the *I*^2^ statistic, with thresholds of 25%, 50%, and 75% representing low, moderate, and high heterogeneity, respectively.

Sensitivity analyses for mortality outcomes were conducted using Cochran Q test across the following subgroups: study design (RCT vs observational), indication for anticoagulation (AF and VTE), follow-up duration (≤6 vs >6 months) and cancer type (solid only vs solid and hematologic malignancies). Post hoc, we also performed additional sensitivity analyses for these subgroups stratified by study design (RCT vs observational) given the observed differences in mortality outcomes between RCTs and observational studies.

## 3 Results

### 3.1 Study characteristics

**Figure 1:**
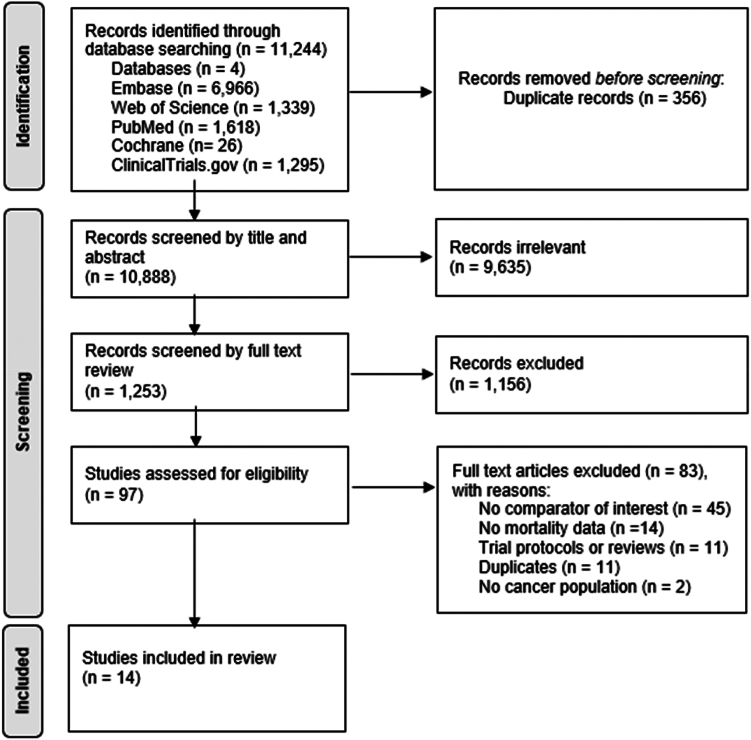
Preferred Reporting Items for Systematic Reviews and Meta-Analyses (PRISMA) flow diagram of the included studies.

Our systematic literature search identified a total of 11,244 studies across 5 databases: Embase (*n* = 6966), Web of Science (*n* = 1339), PubMed (*n* = 1618), ClinicalTrials.gov (*n* = 1295), and Cochrane (*n* = 26) (Figure 1). Prior to screening, 356 duplicate records were removed. Through screening of titles and abstracts, 9635 were excluded. The remaining 1253 articles underwent full-text review, from which 97 studies were selected for eligibility assessment. Of these, 83 were excluded for the following reasons: 45 did not evaluate LMWH or DOACs as comparators, 14 lacked mortality data, 11 were review articles or trial protocols, 11 were duplicate reports, and 2 included populations without cancer. Thus, 14 studies met all inclusion criteria and were included in the final meta-analysis.

The characteristics of the included studies are summarized in Table 1 [4,5,[24], [25], [26], [27], [28], [29], [30], [31], [32], [33], [34], [35]]. The 14 eligible studies were published between 2002 and 2023. Four were conducted in the United States [[24], [25], [26], [27]], 1 in Canada [5], 1 in Finland [28], 1 in the Netherlands [29], 1 in Italy [30], and 1 in France [31]. The remaining 5 were multicenter international studies [4,[32], [33], [34], [35]]. Of the total, 8 were randomized clinical trials [4,5,[30], [31], [32], [33], [34], [35]], and 6 were observational cohort studies [[24], [25], [26], [27], [28], [29]]. The meta-analysis included a total of 70,025 patients, comprising 10,426 females and 59,599 males. Median or mean age for the entire study population was reported in 4 studies [25,26,28,35], while the remaining 10 studies provided age data only for specific subgroups within the population.

**Table 1:** Details of included studies

Reference	Setting, country	Design	Total (*N*)	Follow-up (months), median/mean	Male, *n* (%)	Age	Cancer type	Metastatic, *n* (%)	VKA treated, *n* (%)	Non-VKA–treated, *n* (%)	Risk of bias
Meyer et al. [31], 2002	USA	RCT	146	6	65 (45)	NR	Solid and hematologic	77 (53)	75 (51)	71(49) LMWH	High risk
Lee et al. [4], 2003	Multicenter, international	RCT	676	6	348 (51)	NR	Solid and hematologic	455 (67)	336 (50)	336 (50) LMWH	Some concerns
Hull et al. [5], 2006	Multicenter, Canada	RCT	200	12	102 (51)	NR	Solid and hematologic	83 (42)	100 (50)	100 (50) LMWH	High risk
Lee et al. [32], 2015	Multicenter, international	RCT	900	6	365 (41)	NR	Solid and hematologic	492 (55)	451 (50)	449 (50) LMWH	Some concerns
Amato et al. [30], 2016	Italy	RCT	64	12	29 (45)	NR	Solid and hematologic	NR	32 (50)	32 (50) LMWH	Some concerns
Den Exter et al. [29], 2017	Multicenter, Netherlands	Observational	381	6	203 (53)	NR	Solid and hematologic	NR	234 (61)	147 (39) LMWH	High risk
Melloni et al. [33], 2017	Multicenter, international	RCT	1236	21.6	831 (67)	NR	Solid and hematologic	NR	621 (50)	615 (50) DOAC	Low risk
Alzghari et al. [24], 2018	USA	Observational	127	14.7 (VKA) 6.7 (DOAC) 4.5 (LMWH)	58 (46)	NR	Solid and hematologic	31 (24)	56 (44)	71 (56) LMWH, DOAC	Some limitations
Bauersachs et al. [34], 2018	Multicenter, international	RCT	864	6	353 (41)	NR	Solid and hematologic	NR	440 (51)	412 (49) LMWH	High risk
Chen et al. [35], 2019	Multicenter, international	RCT	640	22.8	423 (66)	77 (72.81) median	Solid and hematologic	NR	331 (52)	309 (48) DOAC	Some concerns
Kinnunen et al. [28], 2019	Finland	Observational	38 747	204	38 747 (100)	60 (55.67) mean	Solid	NR	17 826 (23)	20 921 (77) LMWH/DOAC	Some limitations
Chiasakul et al. [25], 2021	USA	Observational	970	63 (VKA) 57 (LMWH)	4201 (43)	74 (70.80) median	Solid	3720 (38)	4853 (50)	4853 (50) LMWH	Some limitations
Khan et al. [26], 2022	USA	Observational	4274	41	1948 (46)	75 (70.81) median	Solid	1371 (32)	2696 (63)	1348 (37) DOAC	Some limitations
Ryu et al. [27], 2023	USA	Observational	12 298	47.9 (VKA) 34.3 (LMWH and DOAC)	12 298 (97)	NR	Solid and hematologic	2910 (14)	6149 (42)	6149 (58) LMWH, DOAC	High risk

DOAC, direct oral anticoagulant; LMWH, low-molecular-weight heparin; VKA, vitamin K antagonist.

Among included studies VKA was administered to 34,200 patients, LMWH to 18,761 and DOACs to 10,915. One study included 6149 patients treated with either LMWH or DOAC but did not specify the distribution between the 2 groups [27].

The primary indication for anticoagulation was treatment of VTE in 11 studies [4,5,[24], [25], [26], [27],[29], [30], [31], [32],^34^], while 1 study included patients treated for VTE and AF [28] and 2 studies included patients treated for AF only [33,35] (Table 2). Follow-up duration was up to 6 months in 5 studies [4,29,31,32,34] and longer than 6 months in 9 studies [5,[24], [25], [26], [27], [28],^30^,^33^,^35^] (Tables 1 and 2). Eleven studies included patients with either solid or hematologic malignancies [4,5,24,27,[29], [30], [31], [32], [33], [34], [35]], whereas 3 studies included only solid tumors [25,26,28]. Seven studies [4,5,24,26,27,31,32] specified the number of patients with metastatic disease and the remaining 7 studies [[27], [28], [29], [30],[33], [34], [35]] did not specify patient distribution by cancer stage, limiting stage-specific analysis.

**Table 2:** Subgroup analysis for mortality.

Study subgroup	No. of studies	Patient-months	Odds ratio (95% CI)	*I*^2^	*P*
Study design					
RCT	8	VKA: 30,356 Others: 29,593	0.990 (0.866-1.132)	0	.048
Observational	6	VKA: 4,349,543 Others: 4,862,201	0.846 (0.7871-0.915)	81	
Indication for anticoagulation					
AF	2	VKA: 20,960 Others: 20,329	1.009 (0.738-1.380)	74	.098
AF and VTE	1	VKA: 3,636,504 Others: 4,318,094	0.770 (0.671-0.885)	0	
VTE	11	VKA: 722,435 Others: 553,371	0.899 (0.839-0.964)	42	
Follow-up time					
≤6 mo	5	VKA: 9216 Others: 8562	0.976 (0.844-1.130)	0	.120
>6 mo	9	VKA: 4,370,684 Others: 4,883,232	0.856 (0.791-0.926)	75	
Malignancy type					
Solid only	4	VKA: 4,053,602 Others: 4,650,409	0.816 (0.750-0.887)	78	.014
Solid and heme	10	VKA: 326,298 Others: 241,386	0.951 (0.870-1.040)	0	

AF, atrial fibrillation; RCT, randomized clinical trial; VKA, vitamin K antagonist; VTE, venous thromboembolism.

### 3.2 Risk of bias assessment

Of the 8 randomized clinical trials, 3 trials were determined to have a high risk of bias [5,31,34], 4 raised some concerns [4,30,32,35], and 1 was judged to have a low risk [33] (Supplementary Table 2). Of the 6 comparative observational studies included in the meta-analysis, 4 were found to have a moderate risk of bias [[24], [25], [26],^28^], while 2 were classified as high risk [27,29] (Supplementary Table 3).

### 3.3 Mortality analysis

**Figure 2:**
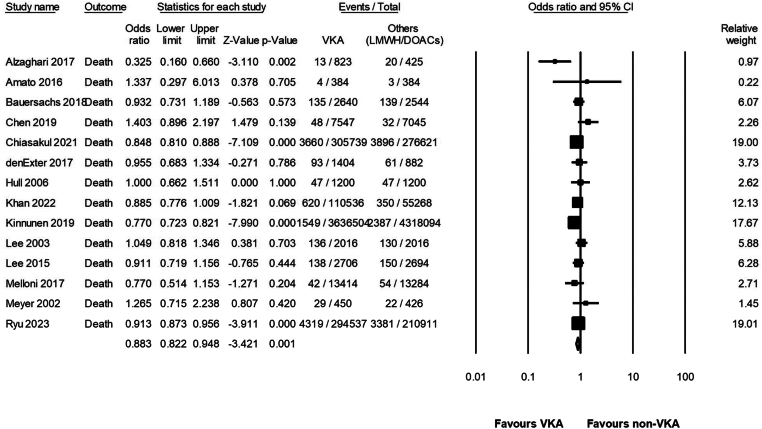
Forest plot showing odds ratios of mortality. DOAC, direct oral anticoagulant; LMWH, low-molecular-weight heparin; VKA, vitamin K antagonist.

The pooled mortality rates in patients treated with VKA was 2.4 per 100 PM (95% CI, 1.9-2.8 per 100 PM; *I*^2^ = 99%; *n* = 14 studies), compared with 2.6 per 100 PM (95% CI, 2.1-3.1 per 100 PM; *I*^2^ = 99%; *n* = 14 studies) in patients treated with LMWH or DOAC (Supplementary Tables 4 and 5). For the group of patients treated with LMWH only, pooled mortality rate was 3.7 (95% CI, 3.0-4.5 per 100 PM; *I*^2^ = 99%; *n* = 10 studies), and for those treated with DOAC, it was 0.5 per 100 PM (95% CI, 0.1-0.8 per 100 PM; *I*^2^ = 99%; *n* = 5 studies). Patients treated with VKA had significant decrease in mortality compared with those treated with non-VKA (Figure 2) (OR, 0.88; 95% CI, 0.82-0.94; *I*^2^ = 65%; *n* = 14 studies) (Table 3). Compared with LMWH specifically, VKA use retained statistically significant survival benefit (OR, 0.82; 95% CI, 0.68-0.99; *I*^2^ = 91%; *n* = 10 studies). Use of DOAC was not associated with a significant difference in mortality compared with VKA (OR, 1.00; 95% CI, 0.98-1.13; *I*^2^ = 74%; *n* = 5 studies).

**Table 3:** Odds ratios of mortality, venous thrombosis, arterial thrombosis, and bleeding.

Outcome	No. of studies	Patient-months	Odds ratio (95% CI)	*I*^*2*^
VKA vs non-VKA				
Mortality	14	VKA: 4,379,900 Others: 4,891,794	0.883 (0.822-0.948)	65
Venous thrombosis	9	VKA: 32,134 Others: 30,474	1.365 (1.042-1.788)	24
Arterial thrombosis	2	VKA: 20,960 Others: 20,329	1.344 (0.7880-2.291)	47
Bleeding	10	VKA: 31,760 LMWH: 30,475	1.103 (0.977-1.246)	10
VKA vs LMWH				
Mortality	10	VKA: 3,953,866 LMWH: 2,801,375	0.828 (0.688-0.996)	91
Venous thrombosis	7	VKA: 11,173 LMWH: 9824	1.265 (0.844-1.897)	64
Bleeding	8	VKA: 10,800 LMWH: 10,146	1.138 (0.965-1.341)	0
VKA vs DOAC				
Mortality	5	VKA: 3,768,824 DOACs: 1,849,927	1.002 (0.980-1.133)	74
Venous thrombosis	3	VKA: 21,784 DOACs: 13,284	1.180 (0.550-2.532)	0
Arterial thrombosis	2	VKA: 20,960 DOACs: 20,329	1.344 (0.788-2.291)	47
Bleeding	3	VKA: 21,784 DOACs: 20,651	1.047 (0.830-1.320)	44

DOAC, direct oral anticoagulant; LMWH, low-molecular-weight heparin; VKA, vitamin K antagonist.

### 3.4 Subgroup analysis of mortality

When stratified by study design, the survival advantage of VKAs remained statistically significant in observational studies (OR, 0.84; 95% CI, 0.78-0.91; *I*^2^ = 81%; *n* = 6 studies). For RCTs, there was no significant difference in mortality when comparing VKA use with other anticoagulant use (OR, 0.99; 95% CI, 0.86-1.13; *I*^2^ = 0%; *n* = 8 studies) (Table 2, Supplementary Figure 1).

Studies with longer periods of follow-up (>6 months) demonstrated a significant survival benefit in the VKA-treated groups (OR, 0.85; 95% CI, 0.79-0.92; *I*^2^ = 75%; *n* = 9 studies), whereas those with shorter periods (≤6 months) showed no difference in mortality between VKA and non-VKA use (OR, 0.97; 95% CI, 0.84-1.13; *I*^2^ = 0%; *n* = 5 studies) (Table 2, Supplementary Figure 2).

A statistically significant reduction in mortality was also observed in studies where VTE was the sole indication for anticoagulation (OR, 0.89; 95% CI, 0.83-0.96; *I*^2^ = 42%; *n* = 11 studies), in a study assessing VTE and AF simultaneously (OR, 0.77; 95% CI, 0.67-0.88; *I*^2^ = 0%; *n* = 1 study) (Table 2, Supplementary Figure 3) and in studies including only patients with solid tumors (OR, 0.81; 95% CI, 0.75-0.88; *I*^2^ = 78%; *n* = 4 studies) (Table 2, Supplementary Figure 4). There was no significant difference in survival with VKA use in studies evaluating patients anticoagulated for AF only (OR, 1.00; 95% CI, 0.73-1.38; *I*^2^ =74%; *n* = 2 studies) and in mixed cancer populations (ie, including both solid and hematologic malignancies) (OR, 0.95; 95% CI, 0.87-1.04; *I*^2^ = 0%; *n* = 10 studies).

Lastly, subgroup analyses (by study duration, indication, and cancer subtype) were conducted separately for RCTs and observational studies. Among RCTs, no statistically significant differences in survival were observed when comparing VKA with non-VKA anticoagulants, regardless of the indication or treatment duration (Supplementary Figures 5 and 6, Supplementary Table 6). In observational studies, VKA likewise conferred no OS benefit compared with non-VKA therapy across different follow-up durations (≤6 months: OR, 0.93; 95% CI, 0.61-1.41; *I*^2^ = 0 %; *n* = 1; >6 months: OR, 0.94; 95% CI, 0.58-1.53; *I*^2^ = 99.03 %; *n* = 5) (Supplementary Figures 7–9, Supplementary Table 7). However, in this setting, a survival advantage was noted for patients treated with VKA for AF or VTE (VTE: OR, 0.99; 95% CI, 0.58-1.69; *I*^2^ = 98.4 %; *n* = 5; VTE ± AF: OR, 0.73; 95% CI, 0.69-0.79; *I*^2^ = 0 %; *n* = 1), as well as in those with solid tumors (solid malignancies only: OR, 0.76; 95% CI, 0.71-0.81; *I*^2^ = 29.2%; *n* = 3; solid with hematologic malignancies: OR, 1.19; 95% CI, 0.63-2.26; *I*^2^ = 87.5 %; *n* = 3).

### 3.5 Secondary outcomes

**Figure 3:**
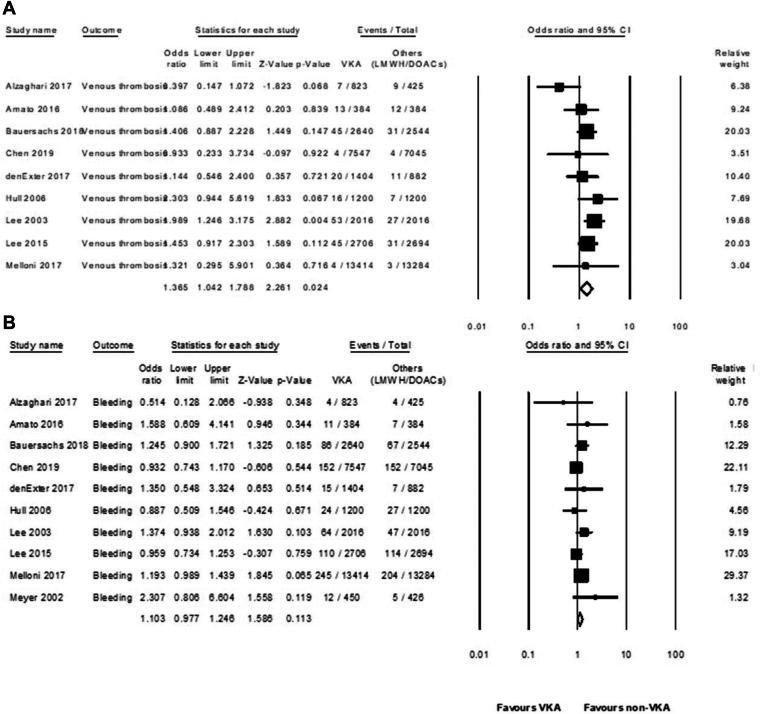
Forest plots showing odds ratios for (A) events of venous thromboembolism and (B) events of bleeding. VKA, vitamin K antagonist.

Pooled rates for venous thrombosis were 1 per 100 PM (95% CI, 0.7-1.2 per 100 PM; *I*^2^ = 96%; *n* = 9 studies) in the VKA group, 1.2 per 100 PM (95% CI, 0.8-1.6 per 100 PM; *I*^2^ = 60%; *n* = 7 studies) in the LMWH group, and 0.05 per 100 PM (95% CI, 0.05-0.1 per 100 PM; *I*^2^ = 60%; *n* = 3 studies) in the DOAC group (Supplementary Tables 4 and 5). Use of VKA was associated with a significantly higher risk of recurrent VTE than non-VKA (OR, 1.36; 95% CI, 1.04-1.78; *I*^2^ = 24%; *n* = 9 studies) (Figure 3A, Table 3). Although the data suggest benefit from the use of non-VKA in venous thrombosis protection, there were no statistically significant differences when comparing VKA with LMWH (OR, 1.26; 95% CI, 0.84-1.89; *I*^2^ = 64; *n* = 7 studies) or DOAC (OR, 1.18; 95% CI, 0.55-2.53; *I*^2^ = 0%; *n* = 3 studies) (Table 3, Supplementary Figures 10 and 11).

For arterial thrombosis, the VKA arm had a rate of 0.3 per 100 PM (95% CI, 0.1-0.6 per 100 PM; *I*^2^ = 90%; *n* = 2 studies) and DOAC had 0.2 per 100 (95% CI, 0.2-0.3 per 100 PM; *I*^2^ = 3 %; *n* = 2 studies) (Supplementary Table 4). There was no statistically significant difference in the risk for arterial thrombosis between patients treated with DOAC compared with VKA (OR, 1.34; 95% CI, 0.78-2.29; *I*^2^ =47%; *n* = 2 studies) (Supplementary Figure 12, Table 3).

Pooled rates for bleeding were 2.3 per 100 PM (95% CI, 1.7-2.9 per 100 PM; *I*^2^ = 91%; *n* = 10 studies) in the VKA arm, 2.1 per 100 PM (95% CI, 1.2-2.9 per 100 PM; *I*^2^ = 87%; *n* = 8 studies) in patients treated with LMWH, and 1.7 per 100 PM (95% CI, 1.1-2.2 per 100 PM; *I*^2^ = 82%; *n* = 3 studies) in patients treated with DOAC (Supplementary Tables 4 and 5). There was no statistically significant difference when comparing bleeding events between VKA- and non-VKA–treated patients (Figure 3B) (OR, 1.10; 95% CI, 0.97-1.24; *I*^2^ = 10%; *n* = 10 studies) (Table 3). When comparing LMWH or DOAC with VKA, the risk of bleeding did not differ significantly (OR, 1.13; 95% CI, 0.96-1.34; *I*^2^ = 0%; *n* = 8 studies; and OR, 1.04; 95% CI, 0.83-1.32; *I*^2^ = 44%; *n* = 3 studies, respectively) (Supplementary Figures 13 and 14). A funnel plot was generated to assess publication bias, with no significant asymmetry observed (Egger’s test *P* = .53; Supplementary Figure 15).

## 4 Discussion

Decisions regarding pharmacologic thromboprophylaxis have traditionally relied on balancing the risks of thrombosis and bleeding, given the limited data on how these agents impact OS. In this meta-analysis, VKA use was associated with improved survival in observational studies, whereas RCTs showed no difference between VKA- and non-VKA–treated patients.

Emerging evidence suggests that a survival benefit associated with VKA use in cancer populations may be multifactorial, potentially extending beyond thromboprophylaxis alone. VKAs, particularly warfarin, may exert antitumor effects through inhibition of coagulation factors involved in tumor progression, as well as interference with noncoagulation pathways critical to tumor biology [36]. This hypothesis is supported by observations of improved survival in warfarin users that persist beyond the duration of active treatment [37]. Preclinical studies have identified a potential mechanism involving inhibition of the growth arrest–specific gene 6–AXL signaling axis. AXL-mediated signaling has been implicated in key oncogenic processes, including epithelial-to-mesenchymal transition, angiogenesis, and immune evasion [[38], [39], [40], [41]]. Overexpression of AXL has been consistently associated with treatment resistance and poor prognosis across several malignancies [[42], [43], [44]]. In animal models of pancreatic cancer, genetic deletion of AXL resulted in an enriched immune microenvironment and prolonged survival [45]. Warfarin has been shown to inhibit growth arrest–specific gene 6–AXL signaling, thereby enhancing the antineoplastic activity of natural killer cells and reducing tumor growth and metastasis [46,47]. These findings suggest that the observed survival benefit of VKAs may, in part, reflect a direct antitumor effect mediated by disruption of pro-oncogenic signaling pathways.

The impact of VKA use on OS, compared with other anticoagulant agents, has been evaluated in multiple RCTs over the past 2 decades. A prior meta-analysis examining the relative effects of LMWH or DOACs vs VKA reported pooled relative risks for all-cause mortality of 1.00 (95% CI, 0.88-1.13) for LMWH vs warfarin (*n* = 1747), and 0.93 (95% CI, 0.71-1.21) for DOACs vs warfarin (*n* = 1031) [48]. These inconclusive results were largely attributed to limited statistical power, underscoring the inherent challenges of detecting survival differences in RCTs. A key limitation of these trials is their relatively short follow-up duration, typically ranging from 3 to 12 months, which might be insufficient exposure time to capture meaningful differences in OS. Additionally, these studies also included cancers of varied types, primary sites, and stages, adding complexity to interpreting the relationship between anticoagulation strategies and survival. To address this limitation, our meta-analysis incorporated observational studies, which allowed for the evaluation of longer-term outcomes. Notably, in a subgroup analyses, these observational studies as well as those with a longer follow-up time revealed a statistically significant survival advantage in patients treated with VKAs compared with those receiving non-VKA anticoagulants.

This study has limitations that must be considered to interpret the results in context. First, the high statistical heterogeneity observed means that pooled estimates should be interpreted with caution. Subgroup analyses suggested that this heterogeneity was primarily driven by observational studies, in which risk of bias may arise from imbalances in baseline characteristics due to the lack of randomization in treatment allocation. Although many of these studies applied matching or statistical adjustments, residual selection bias remains a concern. Second, the study populations were predominantly older (median age, ∼50 years) and largely drawn from Western cohorts, which my limit generalizability. Moreover, key clinical variables that influence survival, such as cardiovascular or metabolic comorbidities and specific anticancer treatments, were not consistently reported. Both observational studies and RCTs also lacked detailed reporting on cancer stage and tumor type, and outcomes were not stratified by disease stage or primary malignancy. These factors are critical determinants of mortality, and their absence limited the ability to perform more detailed subgroup analyses. In addition, total PMs of follow-up were estimated from mean or median follow-up times multiplied by group sample size. While this approach was necessary in the absence of more detailed data, it is important to acknowledge that it represents an approximation. Finally, the predefined study design required inclusion of only those studies that had a VKA arm compared with LMWH, DOAC, or both. This definition limited the feasibility of a network meta-analysis structure and precluded more robust indirect comparisons. However, the pairwise ORs also represented the most transparent and clinically relevant way to assess our primary hypothesis regarding the potential antineoplastic effects of warfarin and its associated survival benefit.

In summary, observational studies suggest that VKA use in patients with cancer may be associated with improved OS. This finding should be interpreted with caution, however, given the high statistical heterogeneity observed and the absence of survival benefit in RCTs. The signal in observational studies may partly reflect longer follow-up durations or a selective effect in patients with solid malignancies. Future studies should aim to clarify the role of VKAs in cancer-associated mortality and determine whether specific tumor types or disease stages derive particular benefit. Importantly, in resource-limited settings, the potential lack of survival advantage with newer agents highlights the continued relevance of cost and availability in guiding anticoagulant selection for patients with cancer-associated VTE.
