# Supplementary material for: Comparing survival with vitamin K antagonists, low-molecular-weight heparin, and direct oral anticoagulants in patients with cancer—a systematic review and meta-analysis
Source: Res Pract Thromb Haemost. 2025 Nov 19;10(1):103268. doi: 10.1016/j.rpth.2025.103268 (PMC12814848; doi:10.1016/j.rpth.2025.103268)
Supplement: Supplementary Material [file mmc1.docx]

**Comparing the outcomes with vitamin K antagonists, low molecular weight heparin and direct oral anticoagulants in patients with cancer- a systematic review and meta-analysis**

**Supplementary Figures**

**Supplementary Figure 1.** Forest plot showing odds ratio of mortality for observational studies and randomized clinical trials.

**
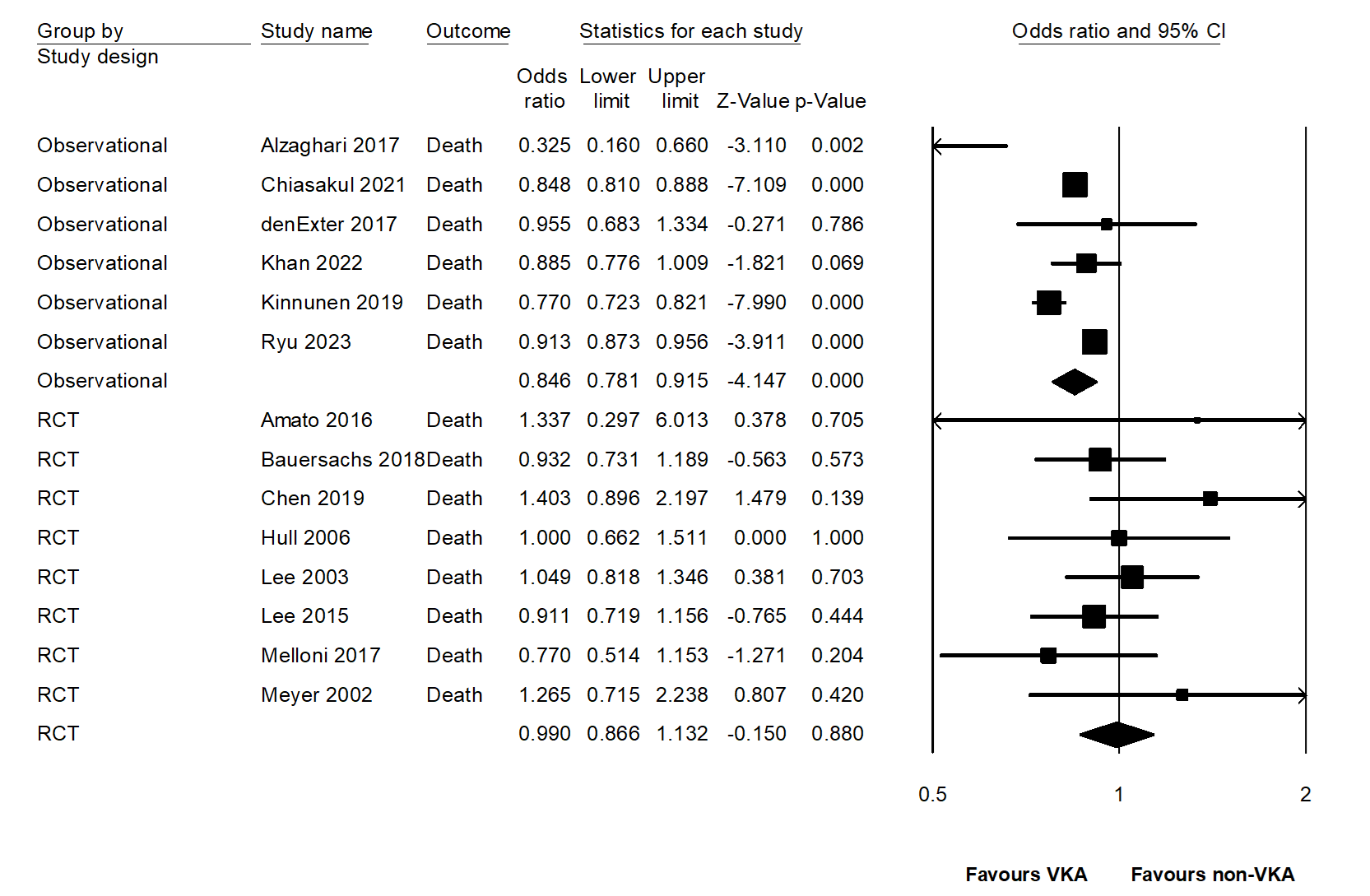
**

**Supplementary Figure 2.** Forest plot showing odds ratio of mortality for different durations of follow up.


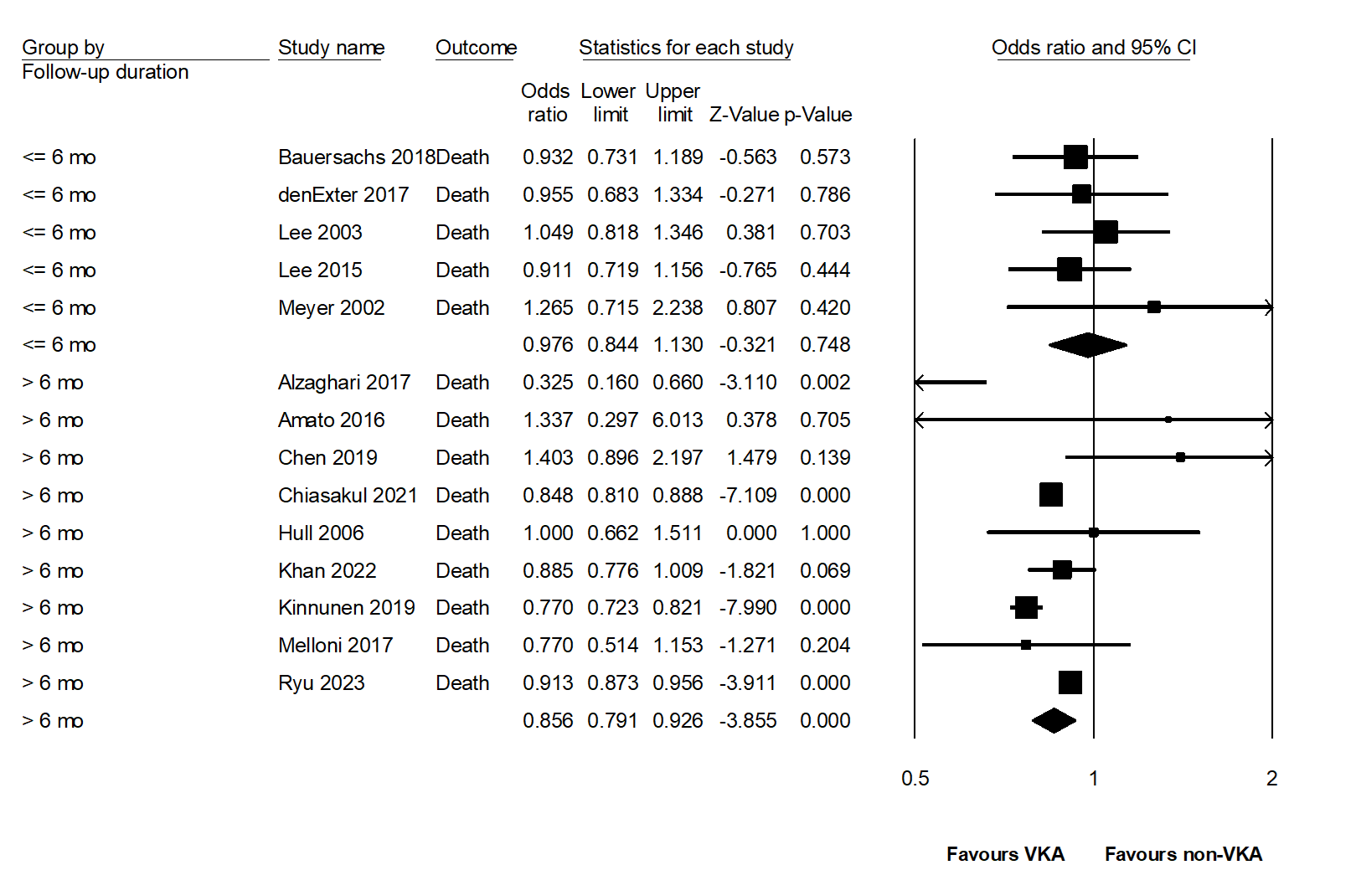


**Supplementary Figure 3.** Forest plot showing odds ratio of mortality for indication of anticoagulation including atrial fibrillation (AF) and venous thromboembolism (VTE).


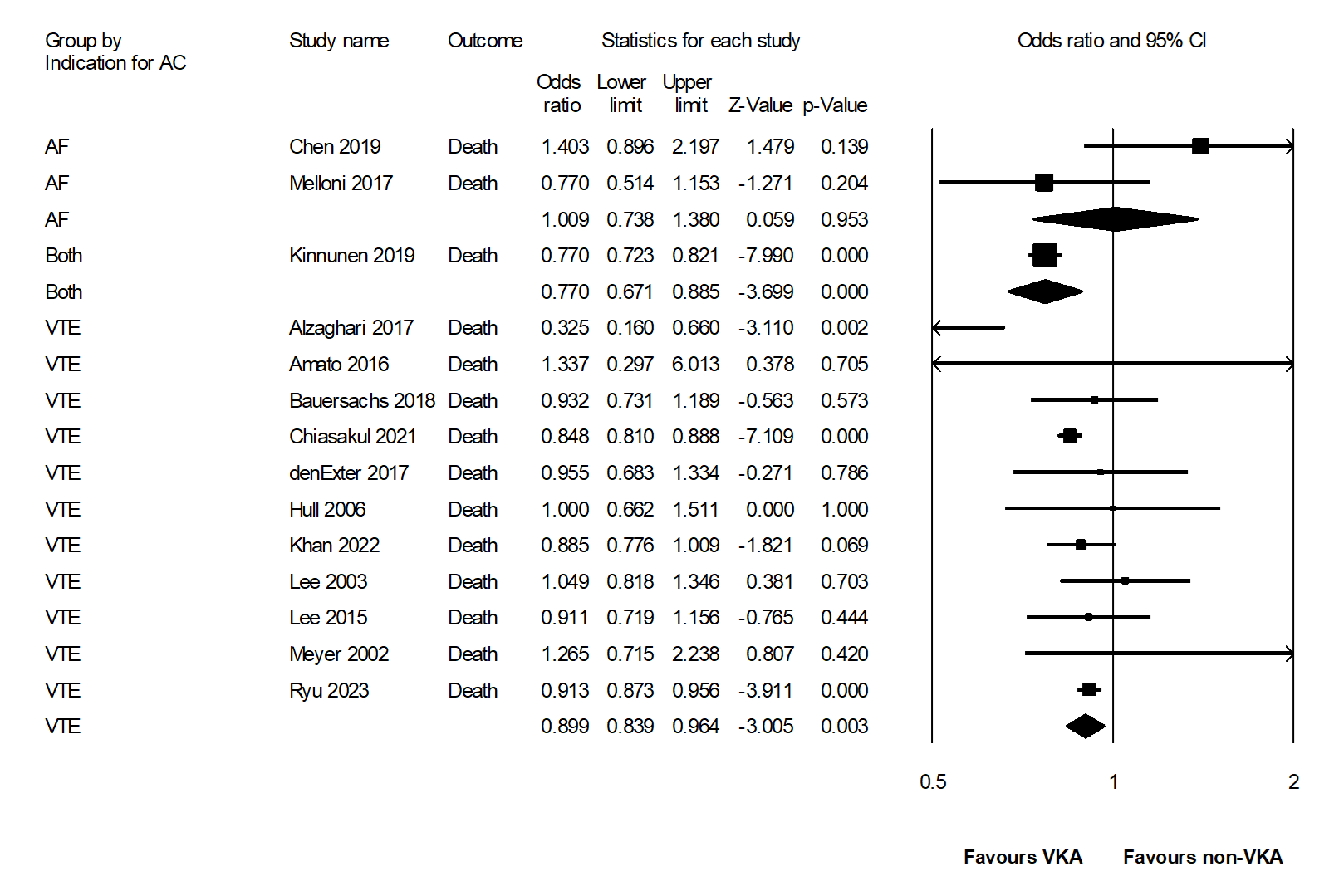


**Supplementary Figure 4.** Forest plot showing odds ratio of mortality for different cancer populations.


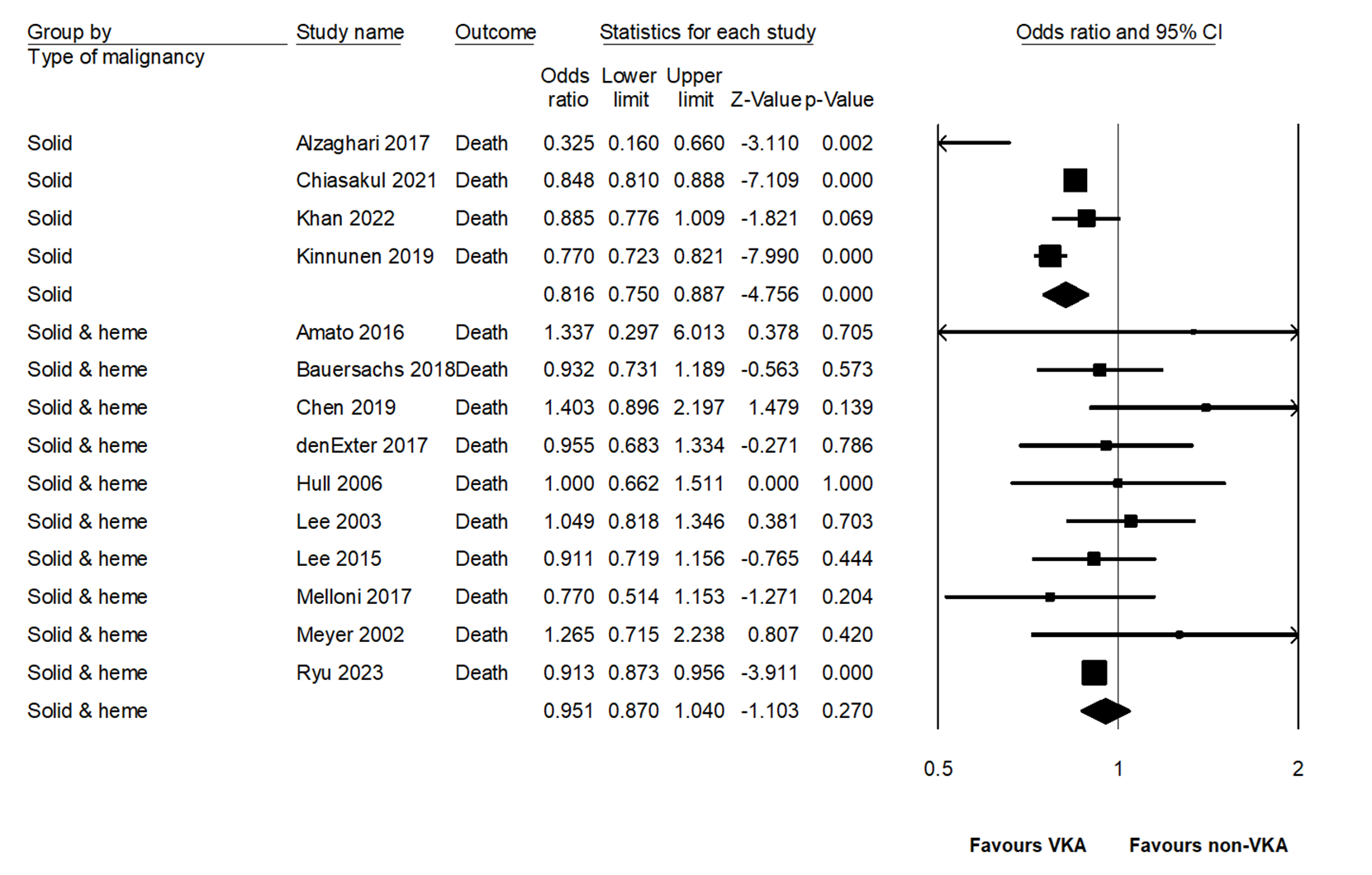


**Supplementary Figure 5.** Forest plot showing odds ratio of mortality for indication of anticoagulation including atrial fibrillation (AF) and venous thromboembolism (VTE) for randomized clinical trials (RCTs) only.

**
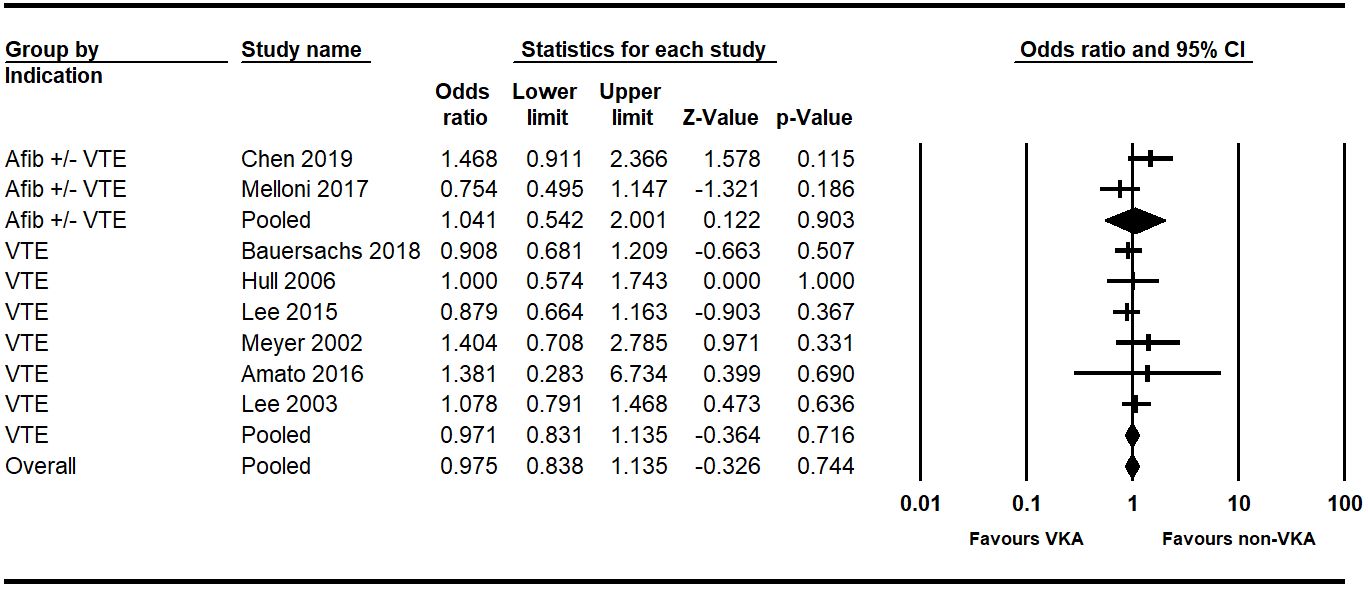
**

**Supplementary Figure 6.** Forest plot showing odds ratio of mortality for different durations of follow up in RCTs only.


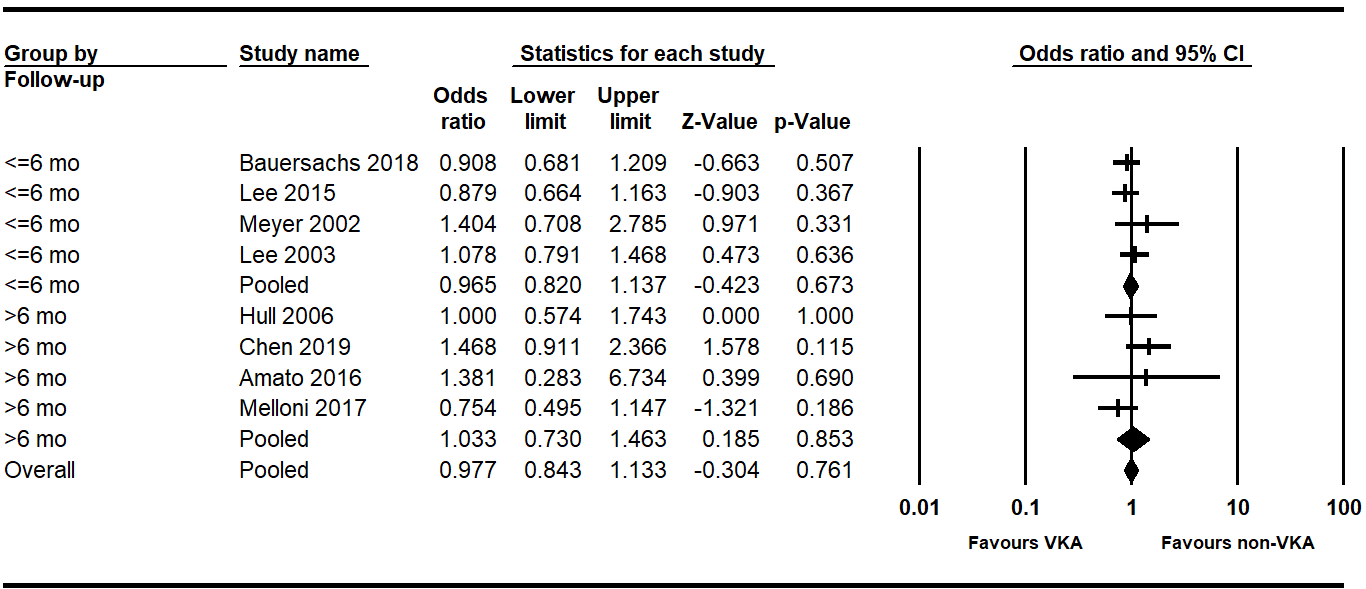


**Supplementary Figure 7.** Forest plot showing odds ratio of mortality for indication of anticoagulation including atrial fibrillation (AF) and venous thromboembolism (VTE) for observational studies only.

**
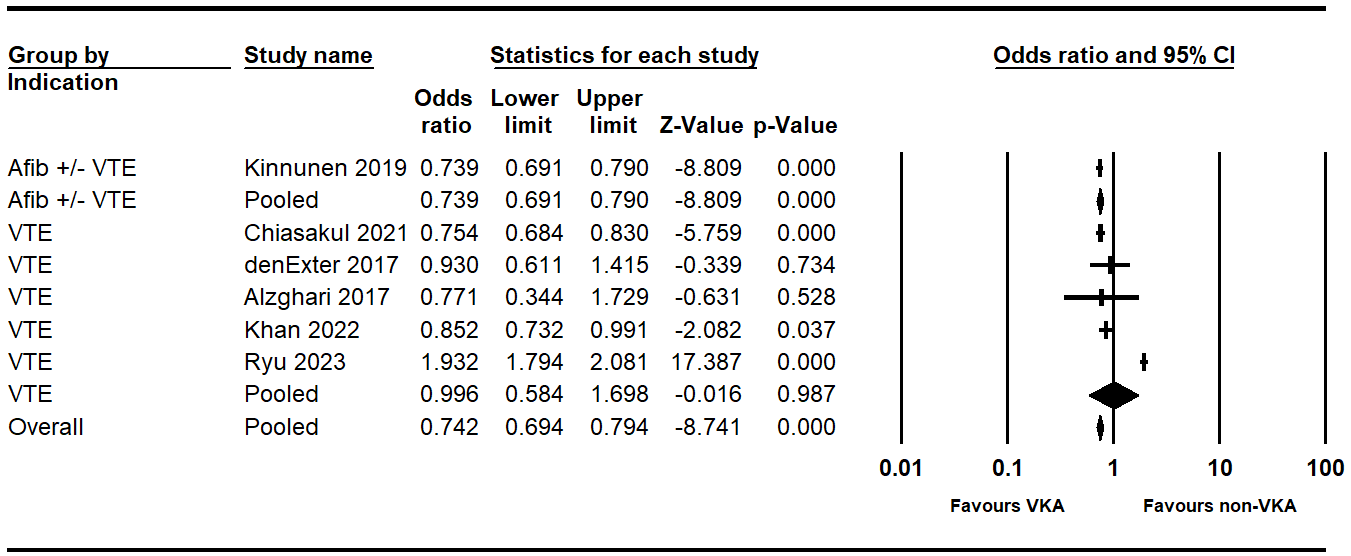
**

**Supplementary Figure 8.** Forest plot showing odds ratio of mortality for different durations of follow up in observational studies only.


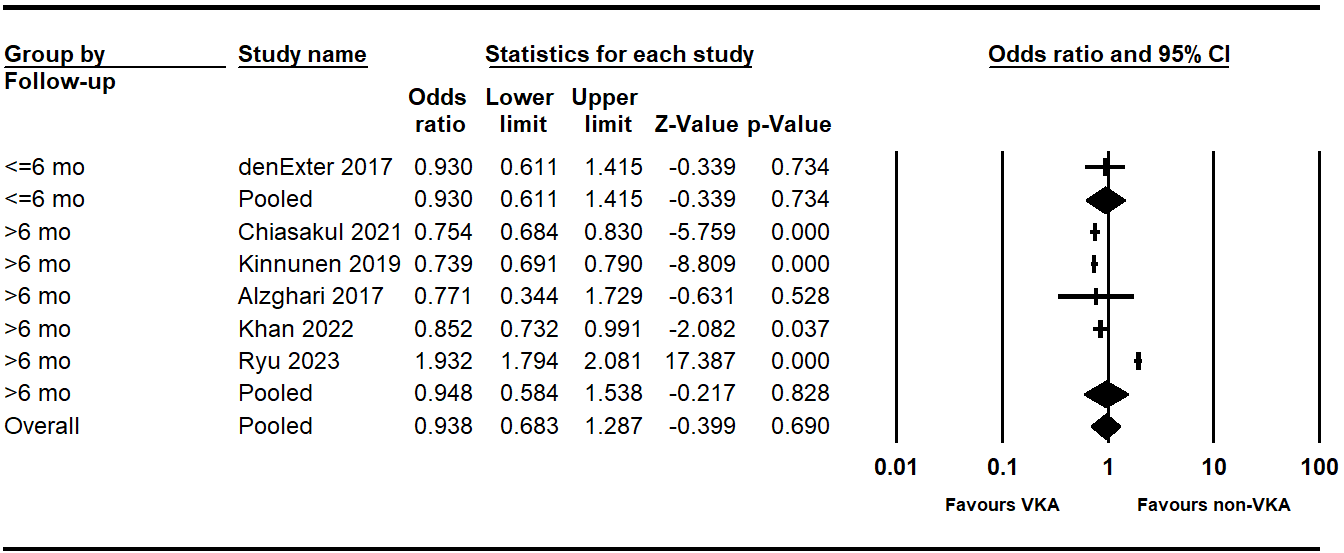


**Supplementary Figure 9.** Forest plot showing odds ratio of mortality for different cancer populations in observational studies only.


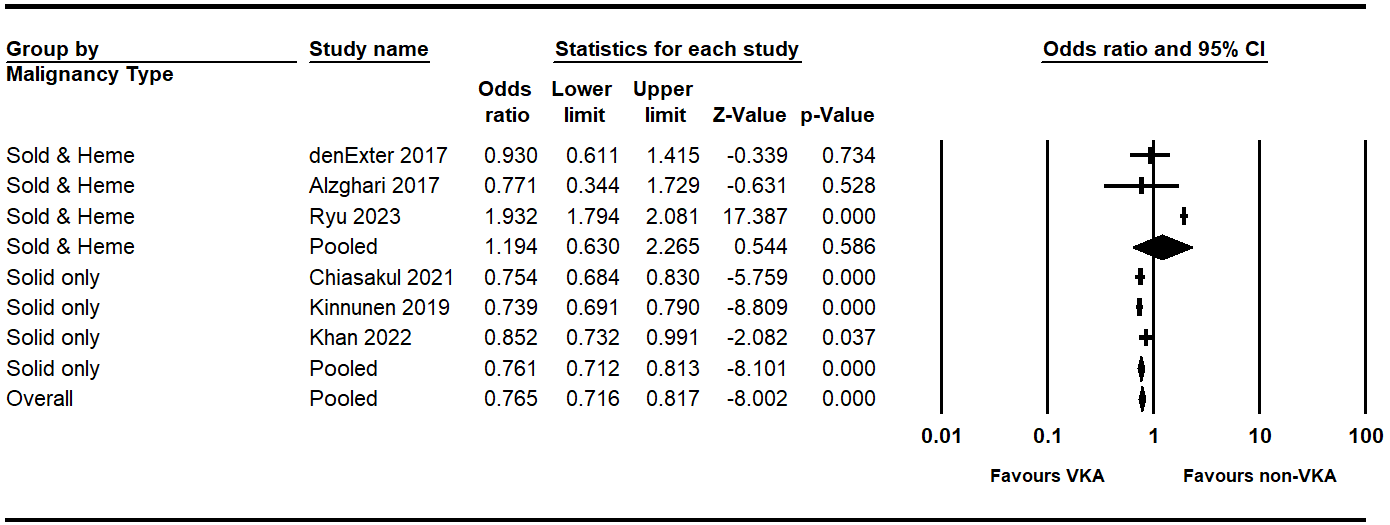


**Supplementary Figure 10.** Forest plot showing odds ratio of venous thromboembolism between VKA and LMWH arms.

**Supplementary Figure 11.** Forest plot showing odds ratio of venous thromboembolism between VKA and DOAC arms.

**Supplementary Figure 12.** Forest plot showing odds ratio of arterial thrombosis.

**
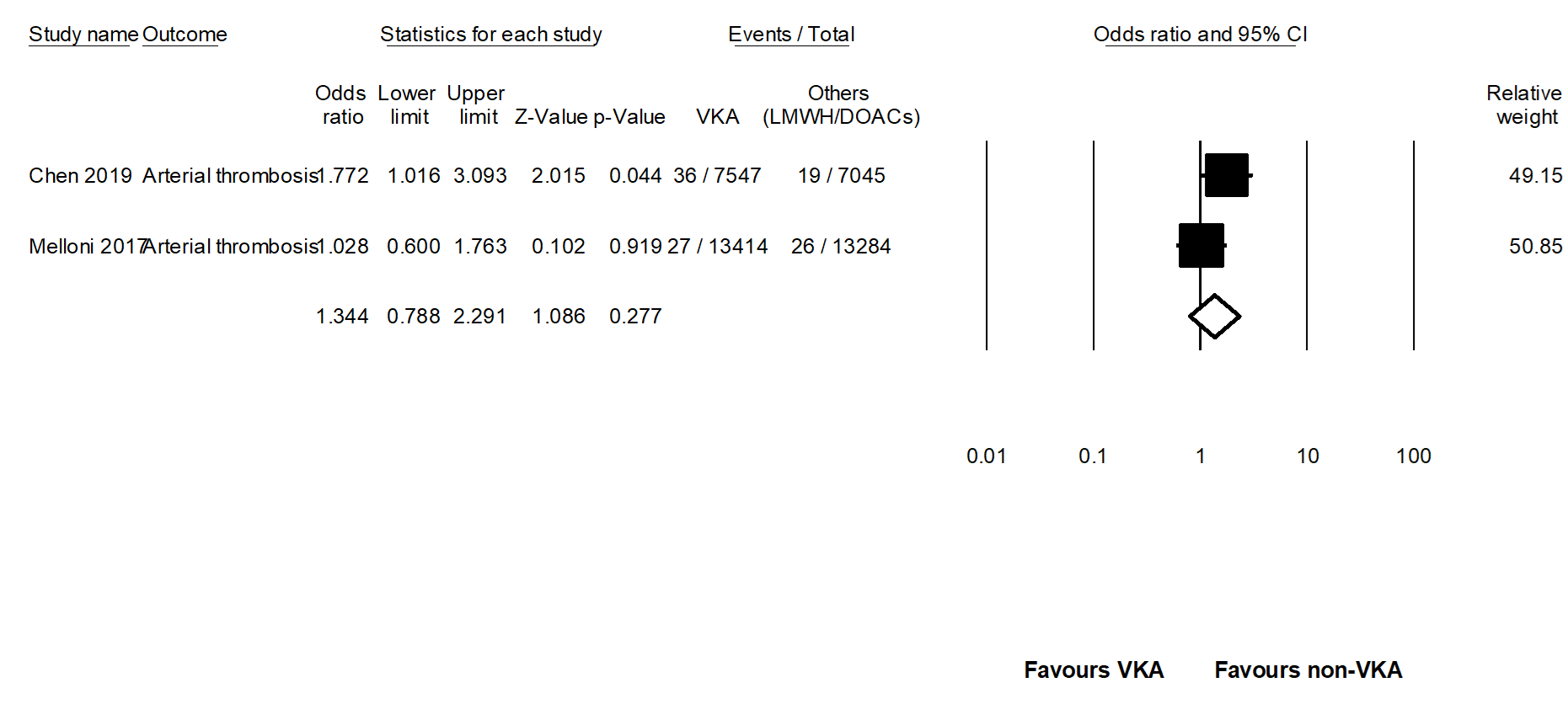
**

**Supplementary Figure 13.** Forest plot showing odds ratio of bleeding between VKA and LMWH arms.

**Supplementary Figure 14.** Forest plot showing odds ratio of bleeding between VKA and DOAC arms.

**Supplementary Figure 15.** Funnel plot for the assessment of publication bias. Egger’s test p-value: 0.53, outcome: mortality.

**
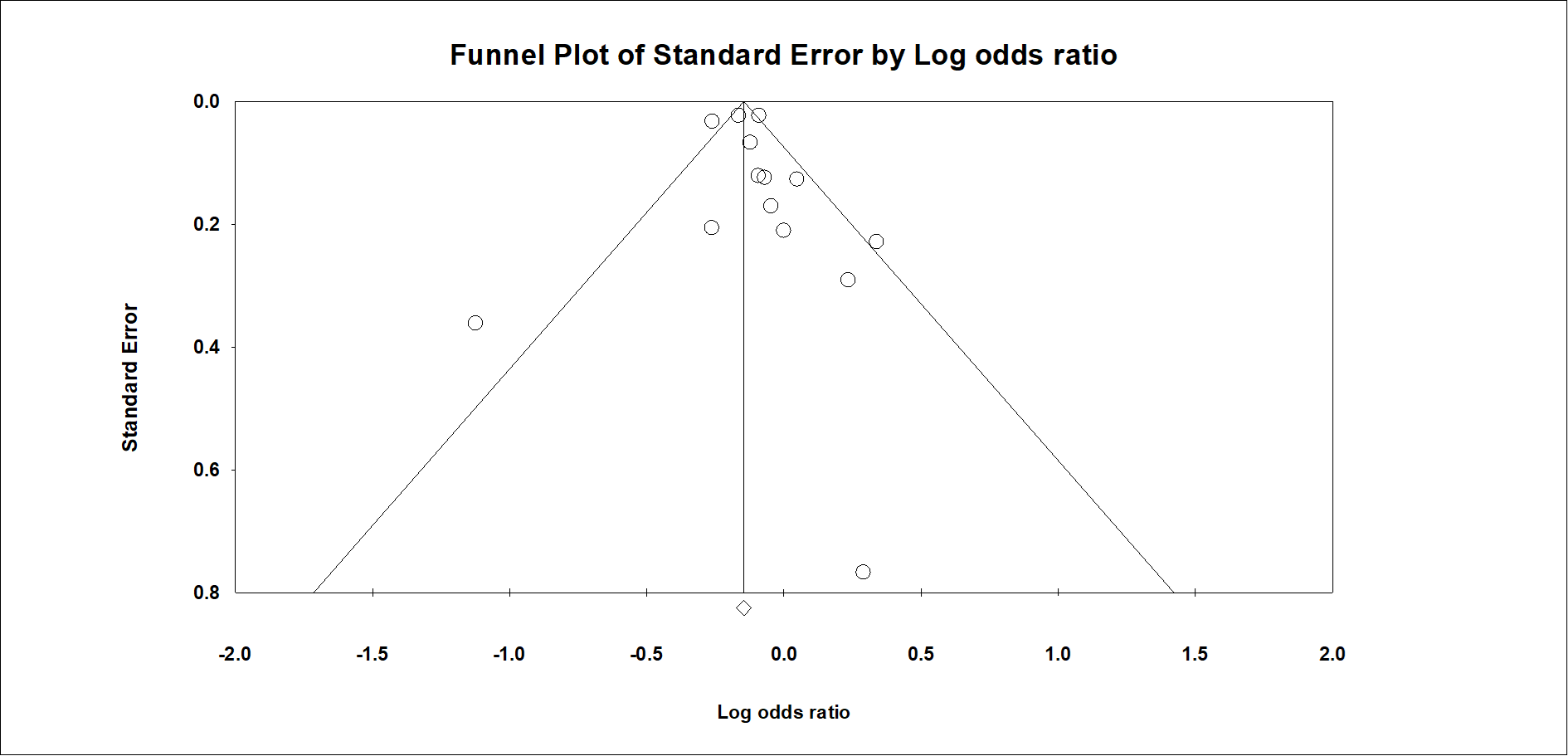
**

**Supplementary Tables**

**Supplementary Table 1.** Data sources and search strategy (date of last search April 10^th^, 2025).

| **Database/Sources** | **Search Strategy** | **Results Retrieved** |
| --- | --- | --- |
| EMBASE | (Tumor:ti,ab OR Neoplasms/exp OR Cancer:ti,ab) AND (('Vitamin K Antagonists':ti,ab OR 'warfarin/therapeutic use*'/exp OR Warfarin:ti,ab OR Anticoagulants/exp OR Anticoagulation:ti,ab OR Anticoagulant:ti,ab OR 'Direct Oral Anticoagulants':ti,ab OR DOACs:ti,ab OR 'Low-Molecular Weight Heparins':ti,ab OR LMWH:ti,ab) AND ( English[lang] )) AND ((term:it) OR (randomized:ti,ab) OR (placebo:ti,ab) OR ('clinical trials as topic'/exp) OR (randomly:ti,ab) OR (trial:ti,ab) NOT (animals/exp) NOT (humans/exp) | 6,966 |
| Cochrane Library, ClinicalTrials.gov | (Tumor:ti,ab OR [mh Neoplasms] OR Cancer:ti,ab) AND (("Vitamin K Antagonists":ti,ab OR [mh ("warfarin/therapeutic" NEXT use*)] OR Warfarin:ti,ab OR [mh Anticoagulants] OR Anticoagulation:ti,ab OR Anticoagulant:ti,ab OR "Direct Oral Anticoagulants":ti,ab OR DOACs:ti,ab OR "Low-Molecular Weight Heparins":ti,ab OR LMWH:ti,ab) AND (English[lang] )) AND (("randomized controlled trial":pt) OR ("controlled clinical trial":pt) OR (randomized:ti,ab) OR (placebo:ti,ab) OR ([mh "clinical trials as topic"]) OR (randomly:ti,ab) OR (trial:ti,ab) NOT ([mh animals]) NOT([mh humans]) | 1,321 |
| PubMed/MEDLINE | ("Tumor"[tiab] OR "Neoplasms"[mesh] OR "Cancer"[tiab]) AND (("Vitamin K Antagonists"[tiab] OR "warfarin/therapeutic use*"[mesh] OR "Warfarin"[tiab] OR "Anticoagulants"[mesh] OR "Anticoagulation"[tiab] OR "Anticoagulant"[tiab] OR "Direct Oral Anticoagulants"[tiab] OR "DOACs"[tiab] OR "Low-Molecular Weight Heparins"[tiab] OR "LMWH"[tiab]) AND ("English"[lang])) AND (("randomized controlled trial"[pt]) OR ("controlled clinical trial"[pt]) OR ("randomized"[tiab]) OR ("placebo"[tiab]) OR ("clinical trials as topic"[mesh]) OR ("randomly"[tiab]) OR ("trial"[tiab]) NOT ("animals"[mesh]) NOT ("humans"[mesh]) | 1,618 |
| Web of Science | \|  \| \| --- \|   (Tumor OR Neoplasms OR Cancer) AND (("Vitamin K Antagonists" OR "warfarin/therapeutic use*" OR Warfarin OR Anticoagulants OR Anticoagulation OR Anticoagulant OR "Direct Oral Anticoagulants" OR DOACs OR "Low-Molecular Weight Heparins" OR LMWH) AND (English)) AND (("randomized controlled trial") OR ("controlled clinical trial") OR (randomized) OR (placebo) OR ("clinical trials as topic") OR (randomly) OR (trial) NOT (animals) NOT (humans) | 1,205 |

**Supplementary Table 2.** Risk of bias for randomized clinical trials included in meta-analysis by Cochrane criteria. Low; low risk, unclear; unclear risk, high; high risk.

| **Cochrane Criteria** | **Meyer**  **2002** | **Lee**  **2003** | **Hull**  **2006** | **Lee**  **2015** | **Amato**  **2016** | **Melloni**  **2017** | **Bauersachs 2018** | **Chen**  **2019** |
| --- | --- | --- | --- | --- | --- | --- | --- | --- |
| Randomization process | unclear | low | low | low | unclear | low | low | low |
| Deviations from intended intervention | unclear | unclear | low | low | low | low | low | low |
| Missing outcome data | high | low | low | unclear | low | low | high | low |
| Measurement of the outcome | low | low | high | low | low | low | low | low |
| Selection of the reported result | low | low | low | low | low | low | low | unclear |

**Supplementary Table 3.** Risk of bias for observational studies included in meta-analysis by MINORS scale. 0; not reported, 1; reported by inadequate, 2; reported and adequate.

| **MINORS Scale** | **denExter 2017** | **Alzghari**  **2018** | **Kinnunen 2019** | **Chiasakul 2021** | **Khan**  **2022** | **Ryu**  **2023** |
| --- | --- | --- | --- | --- | --- | --- |
| Clearly stated aim | 2 | 2 | 1 | 2 | 2 | 2 |
| Inclusion of consecutive patients | 1 | 2 | 2 | 2 | 1 | 0 |
| Prospective collection of data | 0 | 0 | 0 | 0 | 0 | 0 |
| Endpoints appropriate to the aim of the study | 1 | 1 | 2 | 1 | 2 | 2 |
| Unbiased assessment of the study endpoint | 0 | 0 | 0 | 0 | 1 | 1 |
| Follow-up period appropriate to the aim of the study | 1 | 2 | 2 | 1 | 1 | 2 |
| Loss to follow up less than 5% | 1 | 2 | 2 | 2 | 2 | 0 |
| Prospective calculation of the study size | 1 | 0 | 2 | 2 | 0 | 0 |
| Adequate control group | 2 | 2 | 2 | 2 | 2 | 2 |
| Contemporary groups | 2 | 2 | 2 | 2 | 2 | 2 |
| Baseline equivalence of groups | 2 | 2 | 0 | 2 | 2 | 0 |
| Adequate statistical analyses | 1 | 1 | 2 | 2 | 2 | 2 |

**Supplementary Table 4.** Pooled rates for VKA, Non-VKA (including LMWH and DOAC), LMWH and DOAC per patient-months x 100 for rate per 100 patient-months.

| **Outcome** | **VKA** | **Non-VKA** | **DOACs** | **LMWH** |
| --- | --- | --- | --- | --- |
|  | **Pooled rates per patient-month** | **Pooled rates per patient-month** | **Pooled rates per patient-month** | **Pooled rates per patient-month** |
| Mortality | 0.024 (0.019 – 0.028, I^2^ = 99) | 0.026 (0.021 – 0.031, I^2^ = 99) | 0.005 (0.001 – 0.008, I^2^ = 99) | 0.037 (0.030 – 0.045, I^2^ = 99) |
| Venous thrombosis | 0.010 (0.007 – 0.012, I^2^ = 96) | 0.007 (0.005 – 0.009, I^2^ = 93) | 0.000 (0.000 – 0.001, I^2^ = 60)  ** rate is 0.00053*  *(= 0.05 per 100 pt-mo)* | 0.012 (0.008 – 0.016, I^2^ = 60) |
| Arterial thrombosis | 0.003 (0.001 – 0.006, I^2^ = 90) | 0.002 (0.002 – 0.003, I^2^ = 3) | 0.002 (0.002 – 0.003, I^2^ = 3) | --- |
| Bleeding | 0.023 (0.017 – 0.029, I^2^ = 91) | 0.020 (0.015 – 0.025, I^2^ = 88) | 0.017 (0.011 – 0.022, I^2^ = 82) | 0.021 (0.012– 0.029 I^2^ = 87) |

**Supplementary Table 5.** Studies reporting outcomes per arm of anticoagulant treatment.

| **Study Name** | **Reporting Mortality** | **Reporting Venous Thrombosis** | **Reporting Arterial Thrombosis** | **Reporting bleeding** |
| --- | --- | --- | --- | --- |
| Alzghari 2017 | Y (VKA, DOAC, LMWH) | Y (VKA, DOAC, LMWH) | N | Y (VKA, DOAC, LMWH) |
| Amato 2016 | Y (VKA, LMWH) | Y (VKA, LMWH) | N | Y (VKA, LMWH) |
| Bauersachs 2018 | Y (VKA, LMWH) | Y (VKA, LMWH) | N | Y (VKA, LMWH) |
| Chen 2019 | Y (VKA, DOAC) | Y (VKA, DOAC) | Y (VKA, DOAC) | Y (VKA, DOAC) |
| Chiasakul 2021 | Y (VKA, LMWH) | N | N | N |
| denExter 2017 | Y (VKA, LMWH) | Y (VKA, LMWH) | N | Y (VKA, LMWH) |
| Hull 2006 | Y (VKA, LMWH) | Y (VKA, LMWH) | N | Y (VKA, LMWH) |
| Khan 2022 | Y (VKA, DOAC) | N | N | N |
| Kinnunen 2019 | Y (VKA, DOAC, LMWH) | N | N | N |
| Lee 2003 | Y (VKA, LMWH) | Y (VKA, LMWH) | N | Y (VKA, LMWH) |
| Lee 2015 | Y (VKA, LMWH) | Y (VKA, LMWH) | N | Y (VKA, LMWH) |
| Melloni 2017 | Y (VKA, DOAC) | Y (VKA, DOAC) | Y (VKA, DOAC) | Y (VKA, DOAC) |
| Meyer 2002 | Y (VKA, LMWH) | N | N | Y (VKA, LMWH) |
| Ryu 2023 | Y (VKA, non-VKA) | N | N | N |

Y=yes, N=no, VKA=vitamin K antagonists, DOAC=direct oral anticoagulants, LMWH=low molecular weight heparin

**Supplementary Table 6.** Subgroup analysis of RCTs on mortality.

| **Study subgroup** | **N Studies** | **Odds Ratio (95% CI)** | **I^2^** | **P-value** |
| --- | --- | --- | --- | --- |
| **Indication for anticoagulation** | | | | |
| AF +/- VTE | 2 | 1.041 (0.542-2.001) | 76.3 | 0.761 |
| VTE | 6 | 0.971 (0.831-1.135) | 0 |  |
| **Follow-up time** | | | | |
| ≤6 months | 4 | 0.965 (0.820-1.137) | 0 | 0.761 |
| >6 months | 4 | 1.033 (0.730-1.463) | 31.5 |  |

**Supplementary Table 7.** Subgroup analysis of observational studies on mortality.

| **Study subgroup** | **N Studies** | **Odds Ratio (95% CI)** | **I^2^** | **P-value** |
| --- | --- | --- | --- | --- |
| **Indication for anticoagulation** | | | | |
| AF +/- VTE | 1 | 0.73 (0.691-0.79) | 0.00 | 0.234 |
| VTE | 5 | 0.996 (0.584-1.698) | 98.49 |  |
| **Follow-up time** | | | | |
| ≤6 months | 1 | 0.93 (0.611-1.415) | 0.00 | 0.234 |
| >6 months | 5 | 0.948 (0.584-1.538) | 99.03 |  |
| **Malignancy Type** | | | | |
| Solid only | 3 | 0.761 (0.712-0.813) | 29.26 | 0.234 |
| Solid & heme | 3 | 1.194 (0.63-2.265) | 87.50 |  |
